# Identifying children’s environmental health risks, needs, misconceptions, and opportunities for research translation using social media

**DOI:** 10.37349/edht.2024.00011

**Published:** 2024-04-08

**Authors:** Andrew Larkin, Megan MacDonald, Dixie Jackson, Molly L. Kile, Perry Hystad

**Affiliations:** College of Health, Oregon State University, Corvallis, OR 97331, USA

**Keywords:** Social media, environment, children, health

## Abstract

As part of the Advancing Science, Practice, Programming, and Policy in Research Translation for Children’s Environmental Health (ASP^3^IRE) center, machine learning, geographic information systems (GIS), and natural language processing to analyze more than 650 million posts related to children’s environmental health are being used. Using preliminary analyses as examples, this commentary discusses the potential opportunities, benefits, challenges, and limitations of children’s health social media analytics. Social media contains large volumes of contextually rich data that describe children’s health risks and needs, characteristics of homes and childcare locations important to environmental exposures, and parent and childcare provider perceptions, awareness of, and misconceptions about children’s environmental health. Twenty five million unique conversations mentioning children, with likes, views, and replies from more than 33 million X (formerly Twitter) users were identified. Many of these posts can be linked to traditional environmental and health data. However, social media analytics have several challenges and limitations. Challenges include a need for interdisciplinary collaborations, selectivity and sensitivity of analytical methods, the dynamic, evolving communication methods and platform preferences of social media users, and operational policies. Limitations include data availability, generalizability, and self-report bias. Social media analytics has significant potential to contribute to children’s environmental health research and translation.

## Background

Children’s health and wellness is closely tied to their environment. Numerous diverse environmental exposures have been linked to health issues in children, including adverse birth outcomes, asthma, developmental disorders, endocrine disruption, and cancers [1]. For example, lead exposure is linked with neurodevelopmental deficits and cognitive impairment [1, 2] and air pollution is associated with respiratory issues including childhood asthma [3, 4]. Exposure to certain built environment factors, such as fast food outlets and lack of safe spaces for physical activity in urban areas, has also been linked to higher rates of obesity among children [5, 6]. Children are uniquely vulnerable to environmental exposures due to critical periods of development and growth when exposures can have increased negative effects [7]. Thus children’s exposure differs compared to adults (e.g., children eat, drink, and breathe more per kilogram of body weight) [8]. Children are dependent upon the perceptions, awareness, and behaviors of adults and childcare providers to protect them from environmental hazards; their early life exposures can influence developmental and chronic disease trajectories that influence health and wellness later in life [9–11].

While parents and childcare professionals have children’s best interests at heart, it is not always possible to provide children with an optimal environment that takes environmental health into consideration. For small populations, public health researchers and practitioners can leverage contextually rich qualitative methods (e.g., ethnographic studies, interviews, and focus groups) to identify environmental conditions, perceptions, and barriers that impede ideal environmental health conditions. Practitioners then develop logic models (frameworks developed using detailed data and expert knowledge of causal relationships) to promote a positive individual, community, or administrative change focused on environmental health. However, qualitative methods are resource-intensive and sometimes geographically unique—thus their use is often limited for smaller homogeneous samples. For understaffed and underfunded health agencies, it is not feasible to implement qualitative studies for large populations repeatedly over time.

Social media records contain large volumes of contextually rich information pertinent to children’s health. For example, X (formerly Twitter) records contain text, imagery, group interactions such as likes, follows, views, replies, and opt-in geofences (approximate geographic locations). Millions of social media posts from parents, educators, healthcare providers, and other social media users interested in children’s health are publicly available. If automated methods can be developed to extract detailed qualitative data from large volumes of social media records, then social media analytics can potentially generate estimates of individual, familial, and community perceptions, behaviors, needs, awareness, and misconceptions about children’s health for all towns and cities in the US.

Forty-seven percent of US adults use social media as a news source, including 17% that rely on X for news [12]. Among those who use X, only 31% get their news from mainstream news sources, while 36% acquire their news from politicians, celebrities, and influencers [13]. Young adults’ trust in social media information is nearly the same as in national news outlets (50% *vs.* 56%) [14]. Outside of high-profile topics such as elections, coronavirus disease 2019 (COVID-19), and vaccines, social media has few safeguards in place for users to evaluate the quality of information in social media posts [15, 16]. This is problematic because 1) the public needs to have accurate information about environmental hazards that affect children’s health and wellbeing, and 2) misinformation easily spreads on social media.

As part of the Advancing Science, Practice, Programming, and Policy in Research Translation for Children’s Environmental Health (ASP^3^IRE) children’s health center at Oregon State University, we developed a workflow for strategically downloading and analyzing US posts related to children’s environmental health. To date, we have downloaded and are currently analyzing 650 million posts (2014–2023). Our preliminary analyses identified several properties of social media with a high probability of making unique contributions to children’s environmental health research. We also encountered challenges to overcome before inferring relationships from social media, and limitations of social media analyses that need to be considered before integrating social media results into public health research and practice.

## Opportunities and benefits

Social media has enormous untapped potential to advance children’s environmental health and research translation. Three key properties that make social media useful for health research include 1) the ability to follow social media users over time, 2) follow conversations and social media interactions, and 3) automated extraction of rich contextual information from large volumes of social media records.

### Follow users over time

Popular social media sites Facebook, X, Instagram, and more recently Mastodon, and Threads are built around creating digital representations of users and allowing users to follow and interact with each other’s accounts over time. Early Facebook and X adopters have timelines dating as far back as 2005 and 2007, respectively. Among a random sample of 100 active X authors in our social media dataset, 29 have been posting on X for five or more years. By following social media users over time, we can capture natural environmental experiments, where an abrupt event such as construction, wildfires, or a violent crime disrupts normal interactions with the local environment. The ability to follow social media users over time is even more powerful for young families. With nearly 20 years of social media history, we can follow millions of familial developments [17], including the struggles families with children encounter from pregnancy through adolescence [18, 19]. The ability to identify and support struggling members of social media communities is also available [20, 21].

### Follow conversations and social media interactions

Social media users interact with each other through replies, views, follows, likes, subscriptions, and other platform functionality methods. These interactions are useful for identifying how children’s health topics are discussed and how perceptions, risk awareness, and (mis)information about children’s health propagate through social media [22]. Large social media platforms such as X are also popular venues for confrontation, where authors with opposing views argue in “digital town halls”. Identifying confrontations and following users over time can help identify communication strategies with a greater likelihood of disrupting children’s health misconceptions or reinforcing accurate children’s health messages. Our children’s health dataset contains more than 25 million unique conversations mentioning children, with likes, views, replies, and follows of more than 33 million X users posting about children.

### Automated extraction of contextually rich information

Post records contain text, imagery, social interactions, and optionally geographic information. Computer science algorithms are available to extract meaningful contextual information from each of these data types. Natural language processing (NLP) methods such as term-frequency-inverse document frequency [23, 24], combined with deep learning models (e.g., Chat Generative Pre-trained Transformer) can identify exposures, perceptions, behaviors, and social relationships from the text. Similarly, deep learning models for analyzing images can extract valuable information from images. Information that can be extracted from images includes identifying environmental objects and mapping urban environment composition estimating children’s age group(s)/development stage(s) [25, 26], and perceived beauty, nature quality, and safety at photograph locations [27]. NLP and deep learning models have high throughputs for automated large-scale data processing. For example, our deep learning models are capable of processing more than 250 million posts/week using one workstation computer. It is now possible to extract large volumes of contextual data from billions of publicly available records relevant to children’s health. These methods applied to relevant social media data greatly increase the potential for integrating perceptions, barriers, and behaviors into large-scale public health research (epidemiological studies) and translation (interventions and health promotion campaigns).

Automated methods can also be leveraged to geolocate posts and link them to environmental datasets. The percentage of social media posts that contain geographic coordinates or geofences (approximate locations) is small (approximately 1% for X). However, many posts contain text (e.g., “I’m standing right in the middle of Central Park, NYC!”) that can be matched with named locations using NLP algorithms [28]. Social media author profiles also often include a self-reported home town/city. In a previous study, we georeferenced more than 20 million US posts from a single year to city parks using post text, and more than 70% of nature-related posts to a US census-designated place using self-reported user home town/city [28].

The automated pipeline in the ASP^3^IRE social media project is strongly dependent on previously developed NLP and geographic information systems (GIS) algorithms. Our software stack includes ArcGIS Pro for GIS operations, Adobe Spark and Python for NLP and data analytics, Tensorflow, and PyTorch for deep learning, and Neo4j and PostgreSQL for data storage and retrieval. Additional details of the ASP^3^IRE social media software stack are available at the following GitHub repository: https://github.com/larkinandy/ChildrensHealthSocialMediaASP3IRE.

## Challenges to overcome

### Interdisciplinary collaboration

Extracting and analyzing children’s health information from social media records is a challenging effort that requires technical skills and domain knowledge from multiple disciplines. Each step of developing our automated workflow required collaboration from leading experts in child development, environmental health, and computer science. For example, before downloading posts our child health experts identified child age groups and development stages and child health symptoms and health outcomes; our environmental epidemiologists identified environmental exposures and locations where children’s environmental exposures occur; and our computer scientists developed query logic to selectively download posts from the X application programming interface (API), and design a graph database to store raw X records, social relationships, and georeferenced environmental datasets. The best children’s health social media analytics teams will include experts from children’s health, environmental health, GIS, computer vision, data engineering, and NLP.

### Specificity and sensitivity of analytical methods

Social media datasets are enormous. For example, the historical X archive contains on average more than 500 million posts per day. When including metadata such as likes, views, and geographic information along with raw social media posts, datasets can quickly expand to billions of rows in an organized database. Our Neo4j graph database with 650 million posts contains more than 3 billion nodes and 5 billion edges (connections between nodes). Analytical methods with high sensitivity and selectivity are needed to filter through large volumes of records to produce meaningful, high-quality data.

Identifying posts relevant to children is particularly challenging due to the frequent use of child-related keywords such as “baby”, “boy”, and “girl” when referring to adults. While labeling a set of 88,836 posts to create deep learning models, we found that only 26% of posts that contained a child-related word such as “baby” or “toddler” mentioned or depicted a child (true positive) (Table 1). True positive rates significantly differed between keywords, with the lowest rate for posts that contained the word “girl” (5.9%) and the highest rate for posts that contained the word “preschool” (71.5%). Deep learning architectures called “transformers” have significant potential to capture the entire semantic context of a sentence and predict which posts are relevant to children. Our preliminary deep learning transformer models have 97.9% specificity and 64.8% sensitivity in cross-validation datasets, a markedly improved balance between sensitivity and specificity compared to relying on keywords alone.

### Dynamic preferences of social media users

Numerous studies have shown that social media platform popularity is dynamic and changing over time [29]. Eighty-four percent of adults 18–29 use social media, with Instagram (76%), Snapchat (75%), and TikTok (55%) being the most popular [29]. In contrast, only 45% of adults 65 and older use social media, with only 13%, 2%, and 4% using the same three social media sites [29]. Children’s health social media analyses will need to consider spatial and temporal differences in the underlying social media population. Ideally, analyses would include records from multiple social media platforms to increase generalizability and reduce the impacts of changes in social media platform preference.

## Social media limitations

### Data availability

Social media datasets contain billions of records (trillions of records at the global level). Large volume access to these records through programmable APIs is oftentimes limited for public health research. Until recently academics and non-profit researchers could retrieve up to 10 million posts from historical X records per month. Unfortunately, free academic access to posts is no longer supported. Reddit has also recently discontinued free API access to Reddit posts. TikTok, Instagram, and Facebook offer limited API functionality for querying information about recent posts only. User privacy is understandably cited as one of the primary reasons social media platforms restrict large-scale API queries [30]. For social media sites such as Facebook and NextDoor which expressly advertise the exclusivity of who can view group communications, public health researchers will be unlikely to ever access legally or ethically many of these private group discussions. Viable options may include partnering with social media platforms, where platforms take custom analytics and deep learning models created by public health professionals and return anonymized inferences and summary statistics extracted from the subset of data that can be legally and ethically analyzed.

### Generalizability

Although social media usage is widely popular among the US population in general, not all social media use is equal. For example, 20% of US X users post 98% of all US posts [31]. Further, access to social media prerequisites is unequal. Young rural and homeless Americans have disproportionately less access to social media (and consequently may feel disconnected from their peers and support networks) [32, 33]. Social media analyses based on a single social media platform are likely to have limited generalizability, as social media platform preferences differ by demographics, geographical region, and political affinity [29].

### Self-report bias

Social media users are selective in the information they report. Social media accounts have been called digital personas [34], in which users attempt to create an image of themselves that intentionally differs from their true behaviors, perceptions, and experiences. Analyzes based on these accounts may reinforce popular stereotypes including common misconceptions about body types [35, 36], self-esteem [36], and normal behaviors [37]. Soon it is likely algorithms will be able to detect and adjust for or exclude false digital personas from social media analytics. At present, self-report bias on social media is notably large compared to qualitative data collection methods such as anonymized surveys.

The limitations of social media analytics are significant and must be considered in any children’s health social media study. However, despite these limitations, social media analytics has significant potential to contribute to children’s environmental health research and translation. Many of these limitations can be mitigated with future development and refinement of analytical methods. Social media continues to become more and more integral to communication strategies across the world. The sooner that 1) methods are developed to analyze social media content related to children and 2) academic access to publicly viewable social media records is restored, the sooner we can maximize the benefits and minimize the risks of social media for children’s environmental health.

## Figures and Tables

**Table 1.** Percent of posts with select child-related keywords that mention or depict children

Keyword	True positive (%)

Baby	14.2
Boy	8.0
Girl	5.9
Child	38.0
Childhood	6.3
Pediatric	24.7
Preschool	71.5
Teenager	37.9
Toddler	61.2
Youth	34.3
Overall	26.0

## Data Availability

The datasets described in this study can be downloaded from Twitter (X). Due to X developer agreement restrictions, the authors are unable to directly share X social media posts or make these posts publicly available. The methods described in this manuscript will be publicly available in the GitHub repository (https://github.com/larkinandy/ChildrensHealthSocialMediaASP3IRE).

## Abbreviations

API: application programming interface
ASP^3^IRE: Advancing Science, Practice, Programming, and Policy in Research Translation for Children’s Environmental Health
GIS: geographic information systems
NLP: natural language processing
